# Application of Grey Correlation Model to Key Spreader Identification in Multiplex Networks

**DOI:** 10.3390/e28070780

**Published:** 2026-07-09

**Authors:** Shixiang Sun, Xinjiang Wei, Lewei Dong, Huifeng Zhang, Xin Hu

**Affiliations:** 1 School of Mathematics and Statistics Science, Ludong University, Yantai 264025, China; sunshixiangp@163.com (S.S.); donglewei@ldu.edu.cn (L.D.); zhanghuifeng@163.com (H.Z.); huxinsea@163.com (X.H.); 2 School of Mathematics and Statistics, Taishan University, Taian 271000, China

**Keywords:** multiplex networks, identification, influential spreaders, grey correlation model

## Abstract

This study addresses the limitations of existing key node identification methods for multiplex networks—defined as multi-layer networks where all layers share the same node set. To mitigate the loss of critical inter-layer information and improve the reliability of influencer detection, we propose a grey correlation model-based algorithm. The method integrates three essential attributes of multiplex networks: the importance of each individual layer, the intra-layer importance of each node, and node centrality derived from compressing the multiplex network into a single weighted layer. Grey relational analysis is then employed to fuse these heterogeneous attributes and compute a final node significance score. The proposed algorithm is validated on both synthetic and real-world networks using the SIR epidemic model. Experimental results demonstrate that our approach achieves higher node ranking accuracy than six comparison algorithms, confirming its effectiveness in identifying influential spreaders in multiplex networks. Unlike conventional algorithms that typically rely on single-dimensional information, our method systematically combines multiple attributes of multiplex networks, thereby effectively preserving cross-layer topological information and enhancing the reliability of key node identification.

## 1. Introduction

In real-world settings, human society is embedded in a complex landscape of interconnected networks, including biological networks [1], traffic networks [2], communication networks [3], social networks [4], and others [5,6,7]. Against this background, network security threats have intensified, accompanied by the increasingly widespread propagation of viruses and rumors. To address these challenges, key node identification strategies for complex networks [8,9,10,11,12,13]—such as classical centrality measures including degree centrality (DC), k-shell (KS), H-index (H), betweenness centrality (BC), and closeness centrality (CC)—have been widely adopted to enhance network security, mitigate epidemic outbreaks, and contain information dissemination.

All the aforementioned key node identification algorithms are designed exclusively for single-layer complex networks. In practice, however, many real-world systems exist as multi-layer networks formed by the coupling of mutually interacting single-layer networks, e.g., urban transportation networks [14,15], multi-layer social networks [16], and integrated multi-layer computer and power grid networks [17]. Compared with single-layer networks, multi-layer networks exhibit far more intricate topological structures. Their key node identification requires simultaneous consideration of the intra-layer topology within each individual layer and the inter-layer topological connections across different layers. Consequently, most traditional key node identification algorithms for single-layer networks cannot be directly applied to multi-layer scenarios.

Current research on key node identification in multi-layer networks remains relatively limited. Existing algorithms for this task can be primarily classified into three categories: (1) approaches that aggregate or project a multi-layer network into a single-layer network and then apply classic single-layer algorithms to assess node importance [18,19,20,21], which inevitably incur the loss of critical inter-layer topological information; (2) methods that evaluate node importance based on random walk or shortest path strategies [22,23,24], which suffer from excessively high time complexity and are thus ill-suited for large-scale multi-layer networks; and (3) schemes that first apply single-layer spreader identification algorithms to quantify node importance in each layer separately and then fuse these layer-specific importance values through appropriate data fusion techniques.

Beyond the above three categories, recent years have witnessed a surge of novel centrality measures specifically tailored for multi-layer networks [25,26,27,28]. For instance, Li et al. [25] proposed PRGC, an improved gravity centrality that treats multi-PageRank values as node masses and defines a cross-layer weighted distance metric. Nandi et al. [26] introduced a layer dominance measurement framework combined with a closeness-based layer gravity (CLG) method, demonstrating strong scalability on large multiplex networks. Zheng et al. [27] developed the multiplex multi-attribute Laplacian gravity (MMALG) model, which integrates Laplacian centrality with gravity for more accurate node influence assessment. Additionally, tensor decomposition-based methods (e.g., EDCPTD, Wang et al. [28]) offer complementary perspectives on node importance in multi-layer networks.

Existing key node identification algorithms for multi-layer networks typically consider only single-dimensional information, resulting in poor accuracy, while others are burdened by high computational complexity and thus inapplicable to large-scale networks. To overcome these issues, this paper improves the standard grey correlation model by incorporating three key attributes of multi-layer networks: intra-layer node influence, layer-wise importance, and node importance obtained from projecting the multi-layer network onto a single layer.

The proposed IGC algorithm differs from the existing grey correlation model (GC) in three fundamental aspects. First, GC treats each layer equally, whereas IGC explicitly computes layer importance weights and incorporates them into the sample matrix via multiplication. Second, GC operates solely on layer-wise node importance, while IGC additionally projects the multi-layer network into a single layer to extract global connectivity information (CIi), which is then added to the grey relational degree. Third, unlike most multi-layer centrality methods that rely on parametric models (e.g., PageRank, gravity, or tensor decomposition), IGC employs grey relational analysis—a non-parametric, distribution-free technique—to fuse multi-source information, offering robustness against heterogeneous data scales. To the best of our knowledge, this is the first work that systematically integrates grey relational analysis with layer importance and projection-based centrality for key node identification in multi-layer networks.

The structure of this paper is as follows. Section 2 presents the algorithm description, including the grey correlation analysis model and the proposed scheme. Section 3 provides simulation and performance evaluation, covering metrics and case analyses. Section 4 concludes the paper, followed by references.

## 2. Algorithm Description

### 2.1. Grey Correlation Analysis Model

Grey correlation analysis is a quantitative method for comparing and characterizing the dynamic evolution of systems over time. It assesses the degree of relationship between a reference sequence and comparison sequences by measuring the similarity of their geometric shapes [29]. This method is applicable not only to the comprehensive evaluation of time-varying problems but also to analyzing the influence of multiple factors on system outcomes. Its core implementation consists of constructing a parent sequence according to predefined rules, treating each evaluation object as a subsequence, calculating the correlation degree between each subsequence and the parent sequence, and finally drawing conclusions based on the magnitude of these correlation degrees [30,31,32,33]. The specific computational steps of grey correlation analysis are as follows [34]:

**Step 1: Set the sample data.**

An ideal object refers to an entity that accurately captures the behavioral characteristics of a system, while an evaluation object denotes an element that exerts influence on system behavior. Assume that there are l indicators that act on system behavior, with each indicator containing n elements; the elements of the α-th indicator are denoted as $F_{\alpha }=(f_{\alpha 1},\;f_{\alpha 2},\ldots ,f_{\alpha n})$, α=1,2…,l, and the corresponding sample matrix is defined as follows.

$$F=\left[\begin{array}{cccc} f_{11} & f_{12} & \ldots & f_{1n}\\ f_{21} & f_{22} & \ldots & f_{2n}\\ \vdots & \vdots & \ddots & \vdots \\ f_{l1} & f_{l2} & \ldots & f_{\ln} \end{array}\right]$$

**Step 2: Normalize the sample data.**

The mean normalization method, as shown in Equation (1), is adopted in this paper to obtain the normalized data $F^{\prime }$:

(1) $$f_{\alpha j}^{\prime }=\frac{f_{\alpha j}}{\sum_{j=1}^{n}f_{\alpha j}}$$

**Step 3: Determine the analysis object.**

The evaluation object is each column element in the matrix $F^{\prime }$, that is $\left(f_{1j}^{\prime },f_{2j}^{\prime },\ldots ,f_{lj}^{\prime }\right)$(j=1,2,…,n). Select the ideal object as the largest element in each row, that is $f_{\alpha }=\max\{f_{\alpha j}^{\prime }\}$, α=1,2,…,l, j=1,2,…,n. They form an ideal object vector $X=[f_{1},f_{2},\ldots ,f_{l}]$.

**Step 4: Calculate the correlation coefficient.**

The correlation coefficient between the evaluation object and the ideal object is given by Equation (2):

(2) $$\chi_{\alpha j}=\frac{\underset{\alpha =1,2,\ldots ,l}{\min}\underset{j=1,2,\ldots ,n}{\min}\left|f_{\alpha }-f_{\alpha j}^{\prime }\right|+\rho \underset{\alpha =1,2,\ldots ,l}{\max}\underset{j=1,2,\ldots ,n}{\max}\left|f_{\alpha }-f_{\alpha j}^{\prime }\right|}{\left|f_{\alpha }-f_{\alpha j}^{\prime }\right|+\rho \underset{\alpha =1,2,\ldots ,l}{\max}\underset{j=1,2,\ldots ,n}{\max}\left|f_{\alpha }-f_{\alpha j}^{\prime }\right|}$$

Following the standard practice in grey relational analysis, the resolution coefficient is typically set to ρ=0.5, as it balances the influence of the maximum difference term without distorting the ranking order.

**Step 5: Calculate the degree of association.**

Calculate degree of association by Equation (3)

(3) $$R_{i}=\sum_{\alpha =1}^{l}w_{\alpha }\chi_{\alpha j}$$

Where $R_{i}$ represents the degree of association between the ideal object and the evaluation object. Since each indicator has different importance at runtime, weight values $w_{\alpha }$ are introduced, where $\sum_{\alpha =1}^{l}w_{\alpha }=1,\;w_{\alpha }>0$. The larger $R_{i}$ is, the closer the evaluation object is to the ideal object, the stronger the relationship is, and the more important the index i is.

In regards to the feasibility of the selected method, the core idea of the grey correlation model is to construct a parent sequence according to specific rules, treat each evaluation object as a subsequence, compute the correlation degree between each subsequence and the parent sequence, and then draw conclusions based on the magnitude of these correlation degrees. This paper focuses on key node identification in multiplex networks, where node ranking is achieved by assigning importance values to individual nodes. Since nodes within a network are inherently interconnected and mutually influential, it is feasible to identify key nodes by examining the correlation between each node’s importance and that of others in the network.

### 2.2. Proposed Algorithm

A grey correlation model-based algorithm for identifying influential spreaders in multiplex networks is proposed, with its core design ideas elaborated as follows:

(1) The multiplex network is decomposed into its constituent single-layer networks, and the influence of each node within every single layer is computed individually.

(2) Since step (1) accounts only for the intrinsic influence of nodes while neglecting the relative importance of each layer in the overall multiplex network, a layer importance weight is introduced to compensate for this omission.

(3) The layer influence and node intrinsic influence are then fused using grey correlation analysis.

(4) Because the above processing treats the multiplex network as separate single layers and thus overlooks inter-layer topological information, the multiplex network is projected onto a single-layer network to compute the integrated node influence from a global perspective.

(5) Finally, the node importance values are obtained by synthesizing the three types of attribute information described above.

The specific calculation procedure of the proposed scheme is presented as follows:

Consider multiplex complex network $G^{M}$, M = 1, 2,…, m, in which m represents the number of layers of network. Number of nodes in every layer network is n.

**Step 1:** Calculate the nodes’ importance in each layer of network by using the identification algorithm of influential spreaders by local gravity model ($I=\sum_{j\in \Gamma (i)}\frac{k_{i}k_{j}}{d_{ij}^{2}},\;d_{ij}=\frac{1}{g_{ij}}$) [35]. Construct a sample matrix F. Then, normalize the sample matrix F to obtain the normalized matrix $F^{\prime }$.

**Step 2:** Calculate the importance of each layer network. Firstly, aggregate the multiplex network. Then, assign weight to the edges by using the potential edge weight algorithm, that is, the weight of each edge is $w_{e}=k_{i}k_{j}+ks_{i}ks_{j}$. ($k_{i}$ represents the degree of a node, and $ks_{i}$ represents the k-shell value of a node). Secondly, equally assign the weight of each edge to each layer network, and summarize the edge weight in each layer of the network as $w^{\alpha }=\sum_{e=1}^{l_{\alpha }}w_{e}^{\alpha }$. Finally, the importance of each layer network is calculated as $I^{\alpha }=w^{\alpha }/\sum_{\alpha =1}^{L}w^{\alpha }$.

**Step 3:** Obtain matrix ${F_{1}}^{\prime }$ by combining the influence of each layer network with the nodes’ influence of each layer network (${\left({F_{1}}^{\prime }\right)}_{\alpha j}=F_{\alpha j}^{\prime }+I^{\alpha }$). (One important property of addition is that adding the same constant $I^{\alpha }$ to all nodes within the same layer does not change the relative order of the nodes within that layer. This means that the important nodes within the layer still maintain their advantages, and all nodes in the important layer (with a larger $I^{\alpha }$) will obtain a higher overall score. Thus, in the subsequent grey correlation analysis, the nodes in this layer are more likely to approach the ideal object).

**Step 4:** Take the maximum value of each row in matrix ${F_{1}}^{\prime }$ and form a vector $X=[f_{1},f_{2},\ldots ,f_{L}]$ as the ideal object. Then calculate the correlation coefficient $\chi_{i\alpha }$ between each element in ${F_{1}}^{\prime }$ and the ideal object, where α=1,2,…,L.

**Step 5:** Calculate the correlation degree according to the grey correlation, which is $R_{i}=\sum_{\alpha =1}^{l}\chi_{i\alpha }$;

**Step 6:** Calculate the nodes’ influence in the multiplex network. The multiplex network is projected, and nodes’ influence in the projection network can be obtained by $CI_{i}=k_{i}/\sum_{i=1}^{n}k_{i}$;

**Step 7:** By synthesizing the correlation degree and the nodes’ influence in projection network, the final nodes’ importance can be received by $MI_{i}=R_{i}+CI_{i}$.

The pseudo-codes is shown in Algorithm 1.
**Algorithm 1.** The pseudo-codes of ICG**Input: multiplex network *G* =** (***V, E***), **network layers L, nodes in each layer n**
**Output: the centrality of all nodes**
1: Initialize the information
2: **for each** node *i* and layer l, **do**
3:    calculate $F_{i}^{l}\;=\;{FLGM}_{i}^{l}$ **//calculate node influences in each layer**
4: **end for**
5: $F={\left(F_{i}^{l}\right)}_{L\times n}$ **//Construct sample matrix**
6: $F^{\prime }$
**is obtained by** normalizing sample matrix
7: **for** each e in E, **do**
8:      compute $w_{e}$ by $w_{e}=k_{i}k_{j}+ks_{i}ks_{j}$
9: **end for**
10: **for** each *a* in L, **do**
11:    compute $w^{a}$$\;\mathrm{by}\;w^{\alpha }=\sum_{e=1}^{l_{\alpha }}w_{e}^{\alpha }$
12: **end for**
13: compute $I^{a}$ by $I^{\alpha }=w^{\alpha }/\sum_{\alpha =1}^{L}w^{\alpha }$ **//Calculate the importance of each layer of network**
14: compute $F_{1}^{\prime }$ by $F^{\prime }+I^{\alpha }$
15: **for** each l in L, **do**
16:    $f_{l}=\max(F_{1}^{\prime },[],2)$ //Obtain ideal sample
17: **end for**
18: $X=\left[f_{1},f_{2},\ldots ,f_{l}\right]$
19: **for** each j in V, **do**
20:    compute $\chi_{\alpha j}$ by $\chi_{\alpha j}=\frac{\underset{\alpha =1,2,\ldots ,l}{\min}\underset{j=1,2,\ldots ,n}{\min}\left|f_{\alpha }-f_{\alpha j}^{\prime }\right|+\rho \underset{\alpha =1,2,\ldots ,l}{\max}\underset{j=1,2,\ldots ,n}{\max}\left|f_{\alpha }-f_{\alpha j}^{\prime }\right|}{\left|f_{\alpha }-f_{\alpha j}^{\prime }\right|+\rho \underset{\alpha =1,2,\ldots ,l}{\max}\underset{j=1,2,\ldots ,n}{\max}\left|f_{\alpha }-f_{\alpha j}^{\prime }\right|}$ **//Calculate correlation coefficient**
21: **end for**
22: **for** each node v in V, **do**
23:    compute $R_{i}$ by $R_{i}=\sum_{\alpha =1}^{l}\chi_{i\alpha }$ **//Calculate correlation degree**
24:    compute $CI_{i}$ by $CI_{i}=k_{i}/\sum_{i=1}^{n}k_{i}$ **//Calculate the importance of projection network nodes**
25:    compute $MI_{i}$ by $MI_{i}=R_{i}+CI_{i}$ //Calculate final node importance
26: **end for**
27: Return the centrality of all nodes based on *ICG*

**Figure 1:**
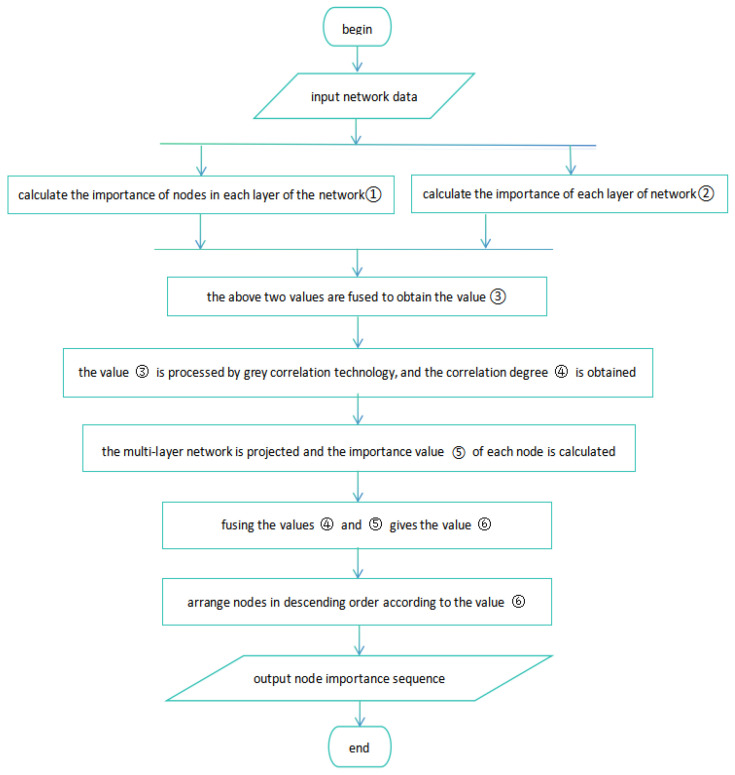
The flow chart of the algorithm.

The flow chart of the algorithm is shown in Figure 1.

## 3. Simulation and Performance Evaluation

### 3.1. Evaluation Index

**Figure 2:**
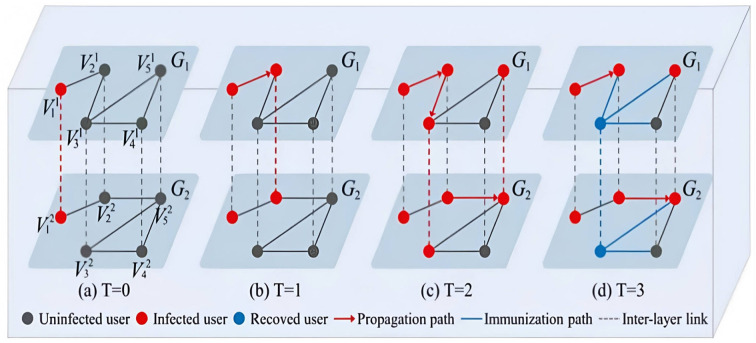
Example of the transmission of disease propagation process in a multiplex networks.

Figure 2 illustrates the transmission mechanism of infectious diseases in a multiplex network [36]. Grey, red, and blue nodes represent susceptible, infected, and recovered (immune) nodes, respectively. Red lines, blue lines, and grey dashed lines denote transmission paths, immune recovery paths, and inter-layer boundaries, respectively.

In a multidimensional multiplex network, a node that is infected in one layer implies that its counterparts in other layers are also infected, that is, at the time of T=0, node $V_{1}^{1}$ is in the infection state as a seed node, and $V_{1}^{2}$ is also in the infection state. The propagation process is as follows: At time T=1, node $V_{1}^{1}$ infects node $V_{2}^{1}$ ($V_{2}^{2}$). At T=2, node $V_{2}^{1}$ infects node $V_{3}^{1}$ ($V_{3}^{2}$), and node $V_{2}^{2}$ infects node $V_{5}^{2}$ ($V_{5}^{1}$). At time T=3, node $V_{3}^{1}$ ($V_{3}^{2}$) returns to immune state.

The following further explains the evaluation:

(1) Propagation Probability

In contrast to a single-layer network, two nodes in a multiplex network may have edges in multiple layers, leading to a higher propagation probability of infectious diseases. The propagation probability for the multiplex SIR model is described by Equation (4):

(4) $$\beta =1-\prod_{\alpha \in S}\left(1-\beta_{\alpha }\right)$$

where $\beta_{\alpha }$ is the propagation probability of network layer α. β is the propagation probability between two nodes in a multiplex network, and S is a set of single-layer networks with edges between two nodes.

(2) Kendall’s Tau Coefficient (τ)

Kendall’s Tau (τ) [37], a measure of correlation between two sequences, is adopted to verify the accuracy of the proposed scheme.

There are two sequences including N elements: $X=(x_{1},x_{2},K,x_{N})$ and $Y=(y_{1},y_{2},K,y_{N})$. If both $x_{i}>x_{j}$ and $y_{i}>y_{j}$, or both $x_{i}<x_{j}$ and $y_{i}<y_{j}$ (i≠j), $\left(x_{i},y_{i}\right)$ and $\left(x_{j},y_{j}\right)$ are said to be concordant two-tuples. If $x_{i}>x_{j}$ and $y_{i}<y_{j}$ or $x_{i}<x_{j}$ and $y_{i}>y_{j}$$\left(x_{i},y_{i}\right)$, then $\left(x_{j},y_{j}\right)$ are discordant. If $x_{i}=x_{j}$ or $y_{i}=y_{j}$, the pair is neither concordant nor discordant. To sum up, the coefficients τ of the two sequences X and Y are defined as Equation (5):

(5) $$\tau =\frac{2(n_{+}-n_{-})}{N(N-1)}$$

where $n_{+}$ is the number of concordant two-tuples and $n_{-}$ is the number of discordant two-tuples. The strength of correlation is measured by the degree to which τ exceeds 0. According to Equation (5), the standard sequence obtained from the SIR model is compared with the sequence generated by other key node identification algorithms. The larger the obtained τ value, the stronger the correlation between the two sequences, indicating that the compared algorithm is more accurate in identifying key nodes.

(3) Monotonicity

To further verify the effectiveness of the proposed method, the sequence monotonicity index $M_{L}$ [38] is introduced to characterize the node discrimination ability of different key node identification algorithms. The greater the diversity of elements in a sequence, the stronger the monotonicity of the sequence. Therefore, a stronger monotonicity of the node sequence generated by an algorithm implies more distinct values in the node importance ranking, indicating superior node discrimination ability. The definition of the sequence monotonicity index is given in Equation (6):

(6) $$M_{L}={\left[1-\frac{\sum_{r\in L}N_{t}(r)(N_{t}(r)-1)}{N(N-1)}\right]}^{2}$$

where L represents sequence and $N_{t}(r)$ is the number of elements sorted by r.

### 3.2. Analysis of Examples

**Figure 3:**
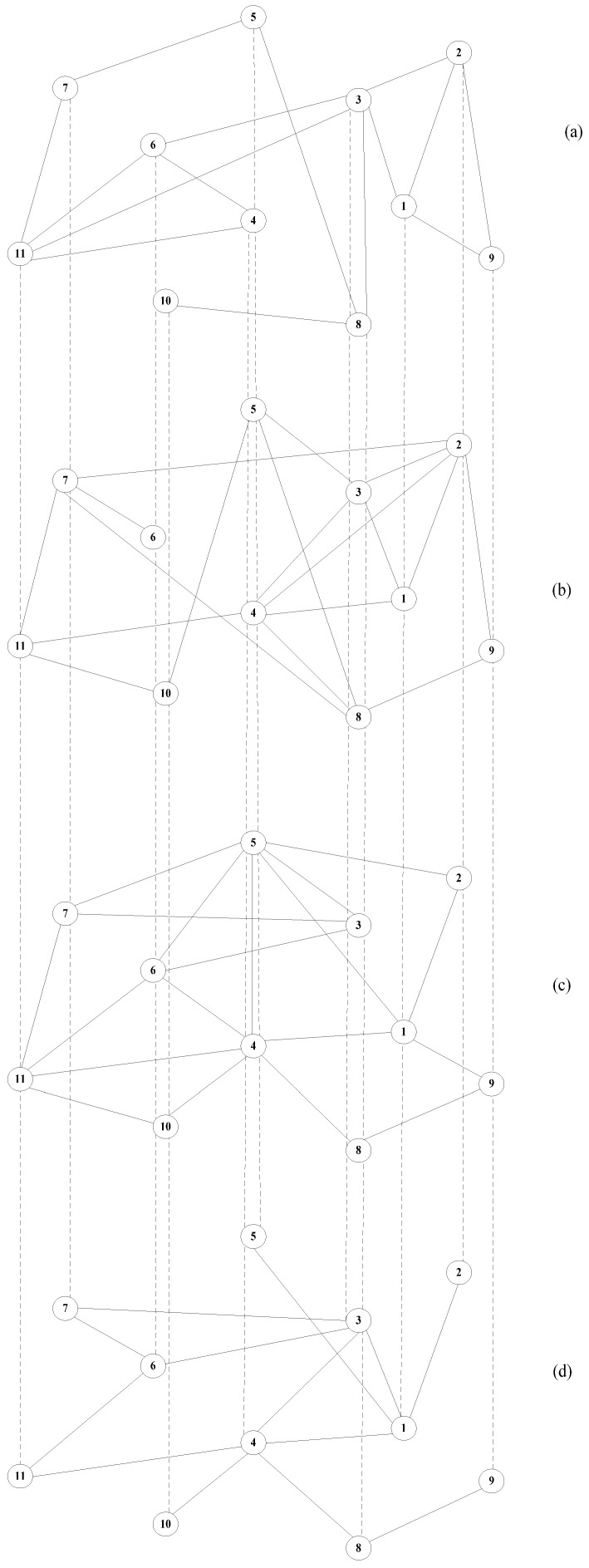
Simple multiplex network’s example. Each layer represents a different connection structure.

The multi-dimensional multiplex network illustrated in Figure 3 is adopted to demonstrate the algorithm flow proposed in this paper, which is composed of four single-layer networks with 11 nodes in each layer.

The steps are as follows:

**Step 1:** The key node mining algorithm of local gravity model is utilized to calculate the node’s influence value in each layer network and then construct the matrix F:

$$F=\left[\begin{array}{ccccccccccc} 534 & 534 & 1055 & 378 & 124 & 600 & 210 & 312 & 348 & 25 & 760\\ 1917 & 3370 & 2816 & 3750 & 1242 & 73 & 1920 & 1434 & 145 & 336 & 1173\\ 3324 & 1026 & 2970 & 6696 & 6930 & 4832 & 2964 & 572 & 496 & 1082 & 4642\\ 540 & 51 & 596 & 760 & 51 & 630 & 334 & 114 & 18 & 58 & 312 \end{array}\right]$$

After that, the sample matrix F is normalized to obtain $F^{\prime }$:

$$F^{\prime }=\left[\begin{array}{ccccccccccc} 1.20 & 1.20 & 2.38 & 0.85 & 0.28 & 1.35 & 0.47 & 0.70 & 0.78 & 0.06 & 1.71\\ 1.16 & 2.04 & 1.70 & 2.27 & 0.75 & 0.04 & 1.16 & 0.87 & 0.09 & 0.20 & 0.71\\ 1.03 & 0.32 & 0.92 & 2.07 & 2.15 & 1.50 & 0.92 & 0.18 & 0.15 & 0.34 & 1.43\\ 1.71 & 0.16 & 1.89 & 2.41 & 0.16 & 2.00 & 1.06 & 0.36 & 0.06 & 0.18 & 0.99 \end{array}\right]$$

**Figure 4:**
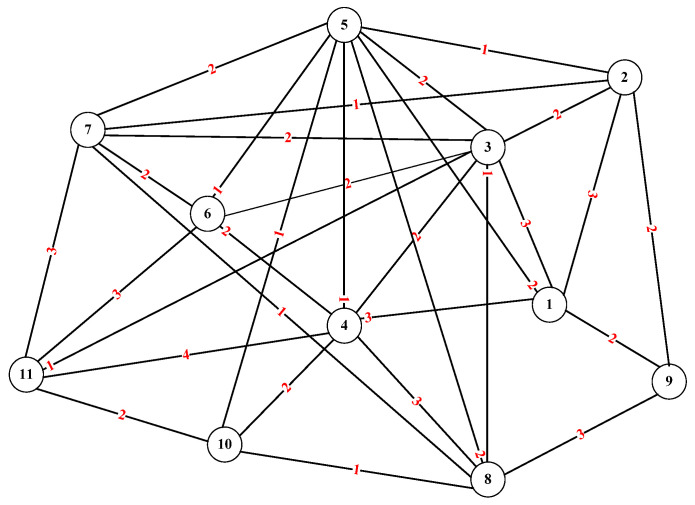
The aggregation network of the multiplex network in Figure 2.

**Step 2:** First, we calculate the importance of each layer in the multiplex network. The multiplex network is first aggregated into a single integrated network, as depicted in Figure 4, where the value assigned to each edge denotes the number of inter-node-connected edges across all layers of the original multiplex network. Taking the edge between Node 1 and Node 2 as an example, if Nodes 1 and 2 are connected by an edge in Layers (a), (b) and (c) of the multiplex network (Figure 2), the corresponding edge value between them in the aggregated network is three.

Secondly, the edges are weighted by $w_{e}=k_{i}k_{j}+ks_{i}ks_{j}$. The weight of each edge is then divided equally among each layer network based on the total number of edges in the aggregate network.

Taking Side 1–2 as an example again, calculations show that the edge weight of Edge 1–2 in the aggregated network is 41. As this edge exists across three layers (a), (b) and (c) of the original multiplex network, the average weight allocated to Edge 1–2 for each individual layer is 41/3.

Thirdly, the weights of all edges in each layer of network are summarized as $w^{\alpha }=\sum_{e=1}^{l_{\alpha }}w_{e}^{\alpha }$. Based on which, the importance of each layer of network is calculated as $I^{\alpha }=w^{\alpha }/\sum_{\alpha =1}^{L}w^{\alpha }$, and the result is shown in Equation (7):

(7) $$I^{\alpha }=\left[0.2321;\;0.2940;\;0.3108;\;0.1631\right]$$

**Step 3:** The matrix ${F_{1}}^{\prime }$ is obtained by combining the importance of each layer network with the importance of nodes.

$$F_{1}^{\prime }=\left[\begin{array}{ccccccccccc} 1.44 & 1.44 & 2.61 & 1.08 & 0.51 & 1.58 & 0.71 & 0.94 & 1.02 & 0.29 & 1.95\\ 1.45 & 2.33 & 2.00 & 2.56 & 1.05 & 0.34 & 1.46 & 1.16 & 0.38 & 0.50 & 1.00\\ 1.34 & 0.63 & 1.23 & 2.38 & 2.46 & 1.81 & 1.23 & 0.49 & 0.46 & 0.65 & 1.74\\ 1.88 & 0.33 & 2.06 & 2.58 & 0.33 & 2.16 & 1.22 & 0.53 & 0.22 & 0.35 & 1.15 \end{array}\right]$$

**Step 4:** The maximum value in each row of the matrix ${F_{1}}^{\prime }$ is used to form a vector $X=[f_{1},f_{2},\ldots ,f_{L}]$ as an ideal object and calculate the association coefficient $\chi_{i\alpha }$ (α=1,2,…,L) between each element in ${F_{1}}^{\prime }$ and the ideal object. The results are shown in Equation (8) and Table 1:

(8) X=[2.61, 2.56, 2.46, 2.58]

**Step 5:** The correlation degree by grey correlation model is calculated:

$$R_{i}=\sum_{\alpha =1}^{l}\chi_{i\alpha }$$

The results are shown in Table 2:

**Step 6:** The multiplex network is projected. Then nodes’ importance in the projection network is calculated: $CI_{i}=k_{i}/\sum_{i=1}^{n}k_{i}$. The values are shown in Table 3.

**Step 7:** By combining the correlation degree with the nodes’ influence in the projection network, the final nodes’ influence can be obtained: $MI_{i}=R_{i}+CI_{i}$. The results are displayed in Table 4.

### 3.3. Experimental Results and Analysis

The proposed algorithm is compared with six other methods—five classical centrality algorithms and one grey correlation method—on four real-world networks and one synthetic network: Padgett, CS-Aarhus, KOC (Krackhardt Office CSS), KAPTAIL (Kapferer Tailor Shop), and BA.

Padgett [39] is a two-layer social relation network compiled by Padgett from historical records, documenting interpersonal connections among 16 Renaissance Florentine families. CS-Aarhus [21] is a five-layer social network consisting of five online and offline relational dimensions (Facebook, leisure, work, cooperation, lunch) among 61 employees of the Computer Science Department at Aarhus University. KOC [40] captures cognitive social structure data from 21 managers at a high-tech machinery manufacturing firm, collected to assess the outcomes of a recent management intervention program. KAPTAIL [41] is a four-layer network that records ten months of instrumental and social interactions among 39 individuals at a sewing shop in Northern Rhodesia. BA denotes a three-layer scale-free network with a total of 500 nodes.

For all five networks, the infection rate in the SIR model is set to the transmission threshold of each network, with a fixed recovery rate of one. The results for each network are derived from 1000 independent experiments.

**In regards to network preprocessing,** the raw data were loaded as unweighted undirected multiplex networks, isolated nodes were retained, and no edge pruning or aggregation was performed prior to the experiments.

To further validate the effectiveness of the proposed method, accuracy experiments were conducted on the five networks with varying infection rates. The infection rate was selected in the range of 0.5β to 1.5β with a step size of 0.1β, where β represents the transmission threshold of the corresponding network’s SIR model. For each network, the infection rate β was set to its epidemic threshold, computed via the method described in [6] (the paper already cites this reference). Specifically, $\beta =1/\lambda_{1}$, where $\lambda_{1}$ is the largest eigenvalue of the supra-adjacency matrix of the multiplex network.

**Figure 5:**
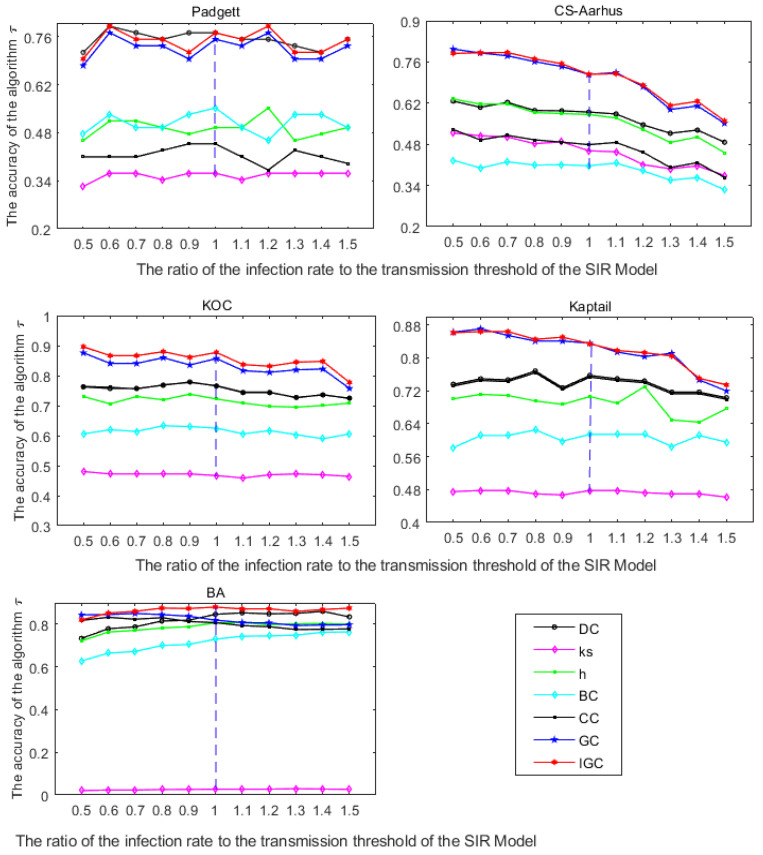
Accuracies of the proposed and benchmark algorithms on 5 multiplex networks under different infection rates.

The experimental results are presented in Figure 5, where the vertical axis represents the correlation coefficient and the horizontal axis represents the ratio of the infection rate to the SIR model’s transmission threshold.

First, the node ranking accuracy of the proposed algorithm was compared against six benchmark methods: Degree Centrality (DC), K-shell (KS), H-index (H), Betweenness Centrality (BC), Closeness Centrality (CC), and the existing grey correlation model (GC). Table 5 reports the ranking accuracy of the proposed algorithm alongside these six methods, while Figure 5 illustrates their accuracy under varying infection rate settings. And Table 6 shows p-values from paired Wilcoxon tests comparing IGC against each baseline (for node ranking accuracy).

The standard deviations are consistently small (typically ≤0.05), indicating that the reported means are stable and the comparisons are reliable.

For each network and each baseline method, we performed a paired Wilcoxon signed-rank test between the 1000 accuracy values of IGC and those of the baseline. The null hypothesis is that the two algorithms have equal median accuracy. The resulting *p*-values are reported in Table 5 *p* (new table). A significance level of α = 0.05 was used.

These results confirm that the observed accuracy advantages of IGC are statistically significant for the vast majority of cases.

From Table 5 and Table 6 and Figure 5, the proposed grey correlation model achieves higher node ranking accuracy than the other six methods across all five multiplex networks. This improvement arises because the presented key node mining framework integrates node importance within each layer, layer importance in the overall network, and node importance in the multiplex structure. In contrast to traditional single-attribute methods, it effectively reduces the loss of critical multiplex information and enhances the precision of node ranking. Next, the sequence monotonicity is utilized to evaluate node resolution of the proposed method. The values are given in Table 7.

It can be observed that the node ordering monotonicity of the proposed IGC algorithm varies across the five multiplex networks. In Padgett, IGC achieves the highest monotonicity (tied with GC); in CS-Aarhus, it is second only to GC; in KOC and Kaptail, it is second only to BC and GC; in BA, it ranks third after BC and CC yet still outperforms GC, DC, ks and h. Overall, IGC exhibits strong node discriminability, though it does not consistently dominate all other methods in monotonicity.

Interestingly, while IGC does not always possess the strongest node discrimination performance, it achieves the highest accuracy in key node mining across all five networks (as shown in Table 5). This demonstrates that there is no inevitable correlation between an algorithm’s key node mining accuracy and its node ordering monotonicity. Compared with monotonicity, using accuracy as an evaluation metric for key node mining algorithms holds greater significance.

Furthermore, the correlation coefficient is employed to quantify the correlation between the proposed model and the comparison methods, with the corresponding values presented in Table 8.

As shown in Table 8, the proposed IGC algorithm exhibits the strongest correlation with the existing GC model. However, unlike the other six comparison algorithms, IGC incorporates multiple attributes of the multiplex network, effectively avoiding the loss of critical network information. This advantage enables IGC to achieve higher node ranking accuracy and monotonicity across all five multiplex networks.

Furthermore, the time complexity of each algorithm is provided to verify the applicability of the proposed IGC algorithm in identifying important nodes in large-scale networks, with detailed results presented in Table 9. The results indicate that the IGC algorithm is more suitable for important node identification in large networks.

Next, the runtime experiments on synthetic multiplex networks with varying sizes is conducted. The experimental setup is as follows:

Number of layers m = 3, average degree $\langle k\rangle$ = 10 per layer.

Node count per layer N ranges from 100 to 10,000.

Reported times are averages of five independent runs with timeout set to 3600 s.

The runtime results are presented in Table 10 below.

The following are observations:

DC and ks are extremely fast (sub-second up to 10,000 nodes), as expected from their linear complexity.

H-index, BC, and CC become impractical for N ≥ 5000, with runtime exceeding one hour due to their cubic or near-quadratic complexity.

GC (existing grey correlation method) scales roughly linearly with N, finishing in about 11 s at N = 10,000.

IGC is approximately 1.8× slower than GC on the largest network (20.6 s vs. 11.2 s), which is consistent with its additional steps (layer importance computation and projection). However, it is still well within practical limits for networks of 10,000 nodes, and it runs more than two orders of magnitude faster than BC/CC.

The runtime results confirm that IGC can handle multiplex networks with up to 10,000 nodes in under 21 s, making it suitable for large-scale applications where classical centrality measures (BC, CC, H-index) are infeasible. For networks beyond 10,000 nodes, further optimizations (e.g., sparse matrix operations, parallelization) can be applied.

**Why does IGC perform better in SIR spreading?**

The SIR model on multiplex networks involves two intertwined processes: intra-layer transmission (local infection) and inter-layer propagation (bridge crossing). A node that is central in only one layer may cause a local outbreak, but its impact remains limited if it cannot spread the infection to other layers. IGC addresses this by jointly evaluating three attributes.

Intra-layer importance (from local gravity) quantifies how quickly a node can infect neighbors within the same layer. Projected centrality counts the total distinct neighbors across all layers, reflecting the node’s potential to carry the infection across different social contexts. Layer importance accounts for the fact that some layers may have higher transmission rates than others.

The grey relational analysis then identifies nodes that are simultaneously strong in all three dimensions by comparing them to an ideal object. This multi-attribute ranking aligns well with the SIR outcome, because an influential spreader must perform well throughout the entire time course-early local infection, cross-layer bridging, and persistence in important layers. Consequently, IGC consistently achieves higher Kendall’s τ than methods that consider only one or two of these aspects.

However, there are limitations in the projection step.

It should be noted that projecting the multi-layer network into a single weighted network, while mitigating the loss of inter-layer topological information, inevitably introduces a certain degree of structural distortion. First, the projection aggregates edges from different layers without preserving layer-specific semantics or topological characteristics, which may be critical in applications where propagation dynamics differ significantly across layers. Second, the simple additive weighting of parallel edges—while computationally convenient—may not accurately reflect the true joint propagation probability, which follows a multiplicative form rather than an additive one. Third, in networks where different layers are densely connected, the projected graph can become overly dense, reducing the discriminative power of $CI_{i}$ and increasing computational cost. Nevertheless, the projected centrality $CI_{i}$ still provides valuable global connectivity information that is entirely absent from the layer-wise analysis alone. The proposed IGC algorithm balances this trade-off by fusing $CI_{i}$ with layer-wise grey relational degrees, allowing the projection to complement rather than replace the fine-grained multi-layer information.

In regards to the application prospects of the IGC algorithm, key node identification in multiplex networks can facilitate a variety of real-world applications, such as the rapid suppression of rumors and virus spread, the prevention of large-scale power outages and catastrophic internet failures, targeted advertising, and the identification of critical proteins.

## 4. Conclusions

In this paper, we propose IGC, a grey correlation model-based algorithm for identifying influential nodes in multiplex networks where all layers share the same node set. The novelty of IGC is threefold: it is the first to integrate grey relational analysis with explicit layer importance weighting, it employs a hybrid multiplicative–additive fusion of intra-layer, inter-layer, and global projection information, and it uses an ‘ideal object’ as a benchmark for node ranking, thereby providing interpretability absent in many existing centrality measures. Experiments on four real-world and one synthetic multiplex network demonstrate that IGC achieves higher node ranking accuracy (Kendall’s τ) than six baseline methods while maintaining strong node discriminability, supporting the claim that IGC effectively avoids the loss of critical inter-layer information within the multiplex setting.

It is important to note that the current IGC algorithm relies on the assumption that all layers have identical node sets; it is not directly applicable to general multi-layer networks with different nodes across layers. Therefore, the conclusions regarding improved reliability and information loss prevention should be understood strictly within the context of multiplex networks. Future work will focus on extending the grey-relational framework to handle heterogeneous node sets across layers and on exploring more efficient fusion strategies.

## Figures and Tables

**Table 1:** The correlation coefficients of nodes in each layer.

Node Number	1	2	3	4	5	6	7	8	9	10	11
(a)	0.50	0.50	1.00	0.44	0.36	0.53	0.38	0.41	0.43	0.34	0.64
(b)	0.52	0.84	0.68	1.00	0.44	0.35	0.52	0.46	0.35	0.36	0.43
(c)	0.51	0.39	0.49	0.94	1.00	0.64	0.49	0.37	0.37	0.39	0.62
(d)	0.63	0.34	0.69	1.00	0.34	0.74	0.47	0.36	0.33	0.35	0.45

**Table 2:** The relevance of nodes in each layer.

Node Number	1	2	3	4	5	6	7	8	9	10	11
Correlation Degree	2.16	2.07	2.86	3.38	2.14	2.27	1.85	1.61	1.48	1.44	2.15

**Table 3:** The nodes’ importance in the projection network.

Node Number	1	2	3	4	5	6	7	8	9	10	11
**Value of CI**	0.08	0.09	0.12	0.12	0.12	0.08	0.09	0.09	0.05	0.06	0.08

**Table 4:** The nodes’ importance in multiplex networks.

Node Number	1	2	3	4	5	6	7	8	9	10	11
**Value of MI**	2.23	2.17	2.98	3.50	2.26	2.34	1.95	1.70	1.53	1.50	2.22
**Rank Number of a Node**	5	7	2	1	4	3	8	9	10	11	6

**Table 5:** Node ranking accuracy (measured by Kendall’s τ coefficient) of the proposed IGC algorithm compared with six baseline methods (DC, ks, h, BC, CC, GC) on five multi-layer networks (Padgett, CS-Aarhus, KOC, Kaptail, BA). Each value is the mean over 1000 independent SIR simulations with the infection rate set to the network-specific epidemic threshold.

Network	DC	ks	h	BC	CC	GC	IGC
**Padgett**	0.7714 (±0.021)	0.3619 (±0.045)	0.4952 (±0.038)	0.5524 (±0.033)	0.4476 (±0.041)	0.7524 (±0.019)	**0.7714** ** (±0.018) **
**CS-Aarhus**	0.5896 (±0.027)	0.4579 (±0.039)	0.5814 (±0.031)	0.4071 (±0.048)	0.4787 (±0.040)	0.7175 (±0.022)	**0.7180** ** (±0.021) **
**KOC**	0.3619 (±0.042)	0.1000 (±0.052)	0.2286 (±0.049)	0.3619 (±0.041)	0.3619 (±0.043)	0.5429 (±0.031)	**0.7524** ** (±0.024) **
**Kaptail**	0.7652 (±0.022)	0.4669 (±0.037)	0.7220 (±0.028)	0.6248 (±0.029)	0.7665 (±0.021)	0.8570 (±0.017)	**0.8772** ** (±0.016) **
**BA**	0.8463 (±0.019)	0.0268 (±0.058)	0.8058 (±0.021)	0.7293 (±0.027)	0.8073 (±0.020)	0.8189 (±0.018)	**0.8801** ** (±0.015) **

**Table 6:** *p*-values from paired Wilcoxon tests comparing IGC against each baseline (for node ranking accuracy).

Network	DC	ks	h	BC	CC	GC	IGC
**Padgett**	**0.482 (n.s.)**	<0.001	<0.001	<0.001	<0.001	<0.001	0.015
**CS-Aarhus**	<0.001	<0.001	<0.001	<0.001	<0.001	<0.001	0.028
**KOC**	<0.001	<0.001	<0.001	<0.001	<0.001	<0.001	<0.001
**Kaptail**	<0.001	<0.001	<0.001	<0.001	<0.001	<0.001	0.004
**BA**	<0.001	<0.001	<0.001	<0.001	<0.001	<0.001	<0.001

(n.s. = not significant, *p* ≥ 0.05). Interpretation: IGC significantly outperforms (*p* < 0.05) all baselines except DC on the Padgett network, where the difference is not statistically significant (*p* = 0.482). On CS-Aarhus, IGC shows a significant but small improvement over GC (*p* = 0.028). On all other networks, IGC is significantly better than every baseline (*p* < 0.001).

**Table 7:** Monotonicity $M_{L}$ (defined in Equation (6)) of the node importance sequences generated by the proposed IGC algorithm and six baseline methods on five multi-layer networks. Higher monotonicity indicates a more diverse ranking with fewer ties.

Network	DC	ks	h	BC	CC	GC	IGC
**Padgett**	0.6249	0.1310	0.4444	0.9437	0.8534	1.0000	**1.0000**
**CS-Aarhus**	0.9093	0.4726	0.8071	0.9837	0.9632	**0.9990**	0.9978
**KOC**	0.9207	0.2827	0.8151	**1.0000**	0.9233	**1.0000**	0.9973
**Kaptail**	0.8950	0.2885	0.8347	**1.0000**	0.8975	**1.0000**	0.9973
**BA**	0.9184	0.0013	0.8192	**1.0000**	0.9897	0.9389	0.9648

**Table 8:** Pairwise Kendall’s τ correlation coefficients between the node rankings produced by the proposed IGC algorithm and those produced by each of the six baseline methods on five multi-layer networks. Values close to 1 indicate strong rank agreement.

Network	DC	ks	h	BC	CC	GC
Padgett	0.7714	0.3619	0.6095	0.4381	0.2762	**0.9810**
CS-Aarhus	0.5754	0.5366	0.5945	0.3623	0.4721	**0.8486**
KOC	0.8136	0.8179	0.8347	0.6338	0.7656	**0.8953**
Kaptail	0.7800	0.5142	0.7611	0.6046	0.7787	**0.9717**
BA	0.8672	0.0303	0.8099	0.7486	0.8351	**0.8863**

**Table 9:** Asymptotic time complexity of the seven evaluated algorithms. N denotes the number of nodes per layer, M the total number of edges across all layers, $\langle k\rangle$ the average degree of a single layer, and *I* the number of iterations (if applicable). The proposed IGC algorithm dominates, and *m* is the number of layers.

Algorithm	Abbreviation	Time Complexity
**Degree Centrality**	DC	O(N)
**k-shell**	ks	O(N + M)
**H-index**	h	O(N^3^)
**Betweenness Centrality**	BC	O(NM + N^2^logN)
**Closeness Centrality**	CC	O(NM + N^2^logN)
**Grey Correlation**	GC	O(mN)
**Algorithm in this paper**	IGC	$O(\mathrm{mN}\langle k\rangle$^2^ + N^2^)

**Table 10:** Running time (in seconds) of all algorithms on multiplex networks of increasing size (m = 3, $\langle k\rangle$ = 10). (Timeout > 3600 s is marked as >3600).

N	DC	ks	h	BC	CC	GC
100	0.002	0.005	0.12	0.85	0.91	**0.15**
500	0.008	0.023	2.31	28.4	30.2	**0.76**
1000	0.015	0.047	15.6	218	235	**1.89**
5000	0.073	0.221	>3600	>3600	>3600	**9.87**
10,000	0.145	0.441	>3600	>3600	>3600	**20.6**

## Data Availability

Data are available upon reasonable request from the corresponding author.

## References

[1] Cao Z.-P., Yang J.-X., Tan Y. (2025). Epidemic spreading on biological evolution networks. Math. Biosci..

[2] Chen Z., Xing Y., Fang K., Lang H., Peng Y., Wang H. (2025). Exploring the impact of extreme weather on urban road traffic networks based on percolation theory. J. Transform. Eng. Part A-Syst..

[3] Yazar A., Danis Z., Cevahir A., Aydin B.B., Atesoglu A., Anuk U. (2025). Scenario-based recommendation approach for wireless communications networks of smart meterss. Telecommun. Syst..

[4] He Q., Fang H., Zhang J., Wang X. (2023). Dynamic opinion maximization in social networks. IEEE Trans. Knowl. Data Eng..

[5] Vinet S., Tannous R., Jennewein T. (2025). A reconfigurable entanglement distribution network suitable for connecting multiple ground nodes with a satellite. EPJ Quantum Technol..

[6] Patel A., Singh B. (2025). Graph convolutional network for structural equivalent key nodes identification in complex networks. Chaos Solitons Fractals.

[7] Liu M., Feng Q., Guo X., Duan D., Lv C., Dui H., Wang Z. (2025). Capability assessment and critical nodes identification in heterogeneous combat networks with multi-functional equipment. Chaos Solitons Fractals.

[8] Guo C.Y., Chen H.C., Wang G.R., Liu S. (2021). A review of importance ranking algorithms and applications of nodes in complex networks. J. Inf. Eng. Univ..

[9] Bonacich P.F. (1972). Factoring and weighting approaches to status scores and clique identification. J. Math. Sociol..

[10] Kitsak M., Gallos L.K., Havlin S., Liljeros F., Muchnik L., Stanley H.E., Makse H.A. (2010). Identification of influential spreaders in complex networks. Nat. Phys..

[11] Hirsch J.E. (2010). An index to quantify an individual’s scientific research output that takes into account the effect of multiple coauthorship. Scientometrics.

[12] Freeman L.C. (1977). A set of measures of centrality based on betweenness. Sociometry.

[13] Freeman L.C. (1978). Centrality in social networks conceptual clarification. Soc. Netw..

[14] Jaber S., Ameli M., Mahdavi S.M.H., Bhouri N. (2025). A methodological framework for resilience as a service (RaaS) in multimodal urban transportation networks. Sustain. Cities Soc..

[15] Zhang J., Chen Y., Wang T., Xie C.-Z.T., Tian Y. (2025). Mixture of spatial-temporal graph transformer networks for urban congestion prediction using multimodal transportation data. Expert Syst. Appl..

[16] Manshad M.K., Meybodi M.R., Salajegheh A. (2022). A new multi-wave continuous action-set cellular learning automata for link prediction problem in weighted multi-layer social networks. J. Supercomput..

[17] Gallotti R., Porter M.A., Barthelemy M. (2016). Lost in transportation: Information measures and cognitive limits in multilayer navigation. Sci. Adv..

[18] Min B., Gwak S.H., Lee N., Goh K.I. (2016). Layer-switching cost and optimality in information spreading on multiplex networks. Sci. Rep..

[19] Chen G.-R. (2013). Problems and challenges in control theory under complex dynamical network environments. Acta Autom. Sin..

[20] Halu A., Mondragon R.J., Panzarasa P., Bianconi G. (2013). Multiplex pagerank. PLoS ONE.

[21] Matteo M., Barbora M., Luca R. (2013). Combinatorial analysis of multiple networks. arXiv.

[22] Jung J.J., Juszczyszyn K., Nguyen N.T. (2007). Centrality measurement on semantically multiplex social networks: Divide-and-conquer approach. Int. J. Intell. Inf. Database Syst..

[23] Sola Conde L., Romance M., Criado R., Flores J., del Amo A.G., Boccaletti S. (2013). Eigenvector centrality of nodes in multiplex networks. Chaos.

[24] Guo Q., Cozzo E., Zheng Z., Moreno Y. (2016). Lévy random walks on multiplex networks. Sci. Rep..

[25] Lv L., Zhang T., Hu P., Bardou D., Niu S., Zheng Z., Yu G., Wu H. (2023). An improved gravity centrality for finding important nodes in multi-layer networks based on multi-pagerank. Expert Syst. Appl..

[26] Nandi S., Maji G., Dutta A. (2025). Identifying vital spreaders in multiplex networks: Measurement of layer dominance and a closeness-based Layer gravity method. J. Supercomput..

[27] Zheng R., Guo Y., Moreno Y. (2025). A new algorithm for identifying influential nodes in multiplex networks. Chaos Solitons Fractals.

[28] Wang D., Wang H., Zou X. (2017). Identifying key nodes in multilayer networks based on tensor decomposition. Chaos.

[29] Sole-Ribalta A., De Domenico M., Gomez S., Arenas A. (2016). Random walk centrality in interconnected multilayer networks. Phys. D Nonlinear Phenom..

[30] Meng W., Chen G., Zeng B. (2026). Assessment of COVID-19’s impact on China’s domestic tourism revenue based on a new grey model with the Hausdorff operator. Grey Syst. Theory Appl..

[31] Li S., Wang Y., Zhang R., Zhang G., Meng W. (2026). A novel seasonal grey multivariate model and its application in China’s new energy vehicle quarterly sales. Grey Syst. Theory Appl..

[32] Guo X., Dang Y., Shen X., Cai Z., Ding S., Huang S. (2026). Spatiotemporal information fusion for urban-agglomeration carbon-emission forecasting: A grey multivariate model with composite city proximity. Grey Syst. Theory Appl..

[33] Guo X., Zhu X., Guo K., Zhong Y., Yang Y. (2026). A novel nonlinear grey Bernoulli model based on recursive regression and pension insurance fund forecast analysis. Grey Syst. Theory Appl..

[34] Liu S.F. (2021). Grey System Theory and Its Application.

[35] Ren T., Sun S., Xu Y., Dimirovski G.M. (2024). Key nodes mining for complex networks based on local gravity model. J. Control. Decis..

[36] Huang X., Chen D., Wang D., Ren T. (2020). Identifying influencers in social networks. Entropy.

[37] Kose E., Burmaoglu S., Kabak M. (2011). Grey relational analysis between energy consumption and economic growth. Grey Syst. Theory Appl..

[38] Chen J., Deng Y., Su Z., Wang S., Gao C., Li X. (2019). Identifying multiple influential users based on the overlapping influence in multiplex networks. IEEE Access.

[39] Breiger R., Pattison P. (1986). Cumulated social roles: The duality of persons and their algebras. Soc. Netw..

[40] Krackhardt D. (1987). Cognitive social structures. Soc. Netw..

[41] Heisler H. (1973). Strategy and transaction in an African factory. Afr. Aff..

